# Human Sperm Remain Motile After a Temporary Energy Restriction but do Not Undergo Capacitation-Related Events

**DOI:** 10.3389/fcell.2021.777086

**Published:** 2021-11-12

**Authors:** Clara I. Marín-Briggiler, Guillermina M. Luque, María G. Gervasi, Natalia Oscoz-Susino, Jessica M. Sierra, Carolina Mondillo, Ana M. Salicioni, Darío Krapf, Pablo E. Visconti, Mariano G. Buffone

**Affiliations:** ^1^ Instituto de Biología y Medicina Experimental (IBYME-CONICET), Buenos Aires, Argentina; ^2^ Department of Veterinary and Animal Science, University of Massachusetts, Amherst, MA, United States; ^3^ Instituto de Biología Molecular y Celular de Rosario (CONICET-UNR), Rosario, Argentina

**Keywords:** glucose, pyruvate/lactate, metabolism, ATP, capacitation, glycolysis, oxidative phosphorylation, sperm motility

## Abstract

To acquire fertilization competence, mammalian sperm must undergo several biochemical and physiological modifications known as capacitation. Despite its relevance, the metabolic pathways that regulate the capacitation-related events, including the development of hyperactivated motility, are still poorly described. Previous studies from our group have shown that temporary energy restriction in mouse sperm enhanced hyperactivation, *in vitro* fertilization, early embryo development and pregnancy rates after embryo transfer, and it improved intracytoplasmic sperm injection results in the bovine model. However, the effects of starvation and energy recovery protocols on human sperm function have not yet been established. In the present work, human sperm were incubated for different periods of time in medium containing glucose, pyruvate and lactate (NUTR) or devoid of nutrients for the starving condition (STRV). Sperm maintained in STRV displayed reduced percentages of motility and kinematic parameters compared to cells incubated in NUTR medium. Moreover, they did not undergo hyperactivation and showed reduced levels of ATP, cAMP and protein tyrosine phosphorylation. Similar to our results with mouse sperm, starvation induced increased intracellular Ca^2+^ concentrations. Starved human sperm were capable to continue moving for more than 27 h, but the incubation with a mitochondrial uncoupler or inhibitors of oxidative phosphorylation led to a complete motility loss. When exogenous nutrients were added back (sperm energy recovery (SER) treatment), hyperactivated motility was rescued and there was a rise in sperm ATP and cAMP levels in 1 min, with a decrease in intracellular Ca^2+^ concentration and no changes in sperm protein tyrosine phosphorylation. The finding that human sperm can remain motile for several hours under starvation due to mitochondrial use of endogenous metabolites implies that other metabolic pathways may play a role in sperm energy production. In addition, full recovery of motility and other capacitation parameters of human sperm after SER suggests that this treatment might be used to modulate human sperm fertilizing ability *in vitro*.

## Introduction

Mammalian sperm acquire fertilization competence in the female tract, in a process known as capacitation (Austin, 1951; Chang, 1951). Capacitation can be mimicked *in vitro* and it is associated with the ability to undergo an agonist-induced acrosomal exocytosis and to develop a distinct type of motility called hyperactivation (Yanagimachi, 1994; Gervasi and Visconti, 2016). Hyperactivated motility is characterized by an asymmetric flagellar beating pattern and allows sperm swimming in viscous environments, detachment from the isthmus reservoir and penetration through the cumulus cells and the *zona pellucida* (ZP) (Ho and Suarez, 2001; Suarez, 2008).

At the molecular level, capacitation requires activation of several signaling pathways, including but not limited to 3′-5′-cyclic adenosine monophosphate (cAMP)-dependent pathways, increase in intracellular pH (pH_i_), changes in intracellular calcium concentration ([Ca^2+^]_i_) and hyperpolarization of the sperm plasma membrane potential (E_m_). Capacitation is also dependent on the activation of several metabolic pathways. Among them, glycolysis and oxidative phosphorylation (OXPHOS) contribute to the synthesis of ATP. The relative contribution of these pathways appears to be species-specific (Visconti, 2012; Du Plessis et al., 2015). In mouse sperm, hyperactivated motility and several capacitation-related events depend on the presence of glucose in capacitation-supporting media (Goodson et al., 2012). Consistently, knock out (KO) mice lacking glycolytic enzymes are sterile (Miki et al., 2004; Danshina et al., 2010). On the other hand, bovine sperm capacitation is inhibited by glucose and these sperm acquire fertilization ability in media containing exclusively Krebs cycle metabolites (e.g. pyruvate and lactate) (Parrish et al., 1989). In human sperm, the main contributor of ATP production is still a matter of debate. While there are several reports indicating that human sperm relies on glycolysis to maintain hyperactivation and capacitation (Rogers and Perreault, 1990; Williams and Ford, 2001; Hereng et al., 2011), there is also evidence indicating that OXPHOS contributes with the energy needed to fuel motility (Ferramosca et al., 2012) and that increased mitochondrial activation, independent of glucose stimuli, occurs during human sperm capacitation (Carrageta et al., 2020).

To study individual contributions of metabolic pathways, we recently developed a methodology in which mouse sperm are first incubated in media devoid of glycolytic and Krebs cycle metabolites. After a certain period of time ranging from 30 to 40 min, mouse sperm stop moving but remain alive. When, in a second step, the sperm incubation media is supplemented with energy nutrients (e.g. glucose and pyruvate), sperm motility is recovered. This method has been named “Sperm Energy Recovery after starvation” and abbreviated with the acronym SER (Navarrete et al., 2019). When compared to sperm persistently incubated in the presence of metabolic nutrients, SER-treated sperm display a significant increase in both hyperactivation and fertilization rates (Navarrete et al., 2019). Most interestingly, the effect of starvation continues after fertilization; SER-treated sperm render higher rates of embryo development to the blastocyst stage. Moreover, when transferred to pseudo pregnant females, thrice the number of pups is born from SER-derived blastocysts in comparison to those obtained with sperm incubated in standard nutrient containing medium.

In this work, we investigated the effect of starvation and rescue on human sperm. Contrary to mouse sperm, human sperm incubated in media devoid of glycolytic and Krebs cycle metabolites did not completely stop moving even after a 27-hour (h) incubation period. In these conditions, the percentages of total and progressive motility were decreased, and sperm velocity was reduced. These results implied the occurrence of ATP production independently of exogenous nutrients in human sperm incubated in starvation. Consistently, addition of mitochondria inhibitors rendered starved sperm completely immotile in few minutes without affecting those that were incubated in the presence of glucose. Similar to the mouse, all human sperm motility parameters were recovered upon addition of nutrients, including the percentage of hyperactive cells, and they underwent several capacitation-related events. Altogether these findings indicate that human sperm are able to synthesize ATP through the catabolic use of endogenous metabolites, at levels sufficient to maintain sperm motility but not capacitation-related events.

## Materials and Methods

### Reagents and Media

Chemicals, unless otherwise indicated, were purchased from Sigma-Aldrich Chemical Co. (St. Louis, MO, United States). Rotenone and antimycin A were from Cayman Chemical (Cat #13,995 and 19,433, respectively; Ann Arbor, MI, United States). The antibodies used were: anti-phospho-protein kinase A substrates (pPKAs) (Cat #9624s; Cell Signaling Technology, Danvers, MA, United States), anti-phosphotyrosine (pY) (clone 4G10; Millipore Corporation, Temecula, United States), anti-β-tubulin (Cat #T4026; Sigma-Aldrich), anti-rabbit and anti-mouse conjugated with horseradish peroxidase (HRP) (Sigma-Aldrich and Vector Laboratories, Inc., Burlingame, CA, United States, respectively), and anti-cAMP antibody (gently provided by Dr. Parlow, National Hormone and Peptide Program, Harbor-UCLA Medical Center, CA, United States). 20-O-monosuccinyladenosine-30,50-cyclic monophosphate tyrosylmethyl ester (TME-cAMP) was radiolabeled with Na^125^I by the method of chloramine-T 90 (specific activity 600 Ci/mmol) as described (Piroli et al., 1992). Fluo-4 AM and pluronic acid were purchased from Invitrogen, Thermo Fisher Scientific (Waltham, MA, United States); while propidium iodide (PI) was from Santa Cruz Biotechnology (Dallas, TX, United States). Biggers-Whitten-Whittingham medium (BWW (World Health Organization, 2010)) was used throughout the study. It consisted of (in mM): 94.3 NaCl, 4.78 KCl, 1.2 MgSO_4_, 1.7 CaCl_2_, 1 KH_2_PO_4_, 5.5 glucose, 0.27 Na-pyruvate and 25 Na-lactate. This medium was called “NUTR”. Medium devoid of glucose, pyruvate and lactate was used for starving condition (“STRV”). For SER experiments, 2x NUTR medium was added to starved sperm, which contained the same components of NUTR but with double amount of the energy sources (11 mM glucose, 0.54 mM Na-pyruvate and 50 mM Na-lactate). One hour before use, media were supplemented with penicillin G, 25 mM NaHCO_3_ and 0.5% bovine serum albumin (BSA; Cat #A7960; Sigma-Aldrich) and placed at 37°C in a 5% (v/v) CO_2_ atmosphere. All media were used at pH = 7.4.

### Semen Samples, Sperm Selection and Capacitation

Semen samples used in the study were provided by normozoospermic volunteers, and obtained under donors’ written consent. Protocols were reviewed and approved by the Ethics Committee of the Instituto de Biología y Medicina Experimental, Buenos Aires (Ref: CE 001/April 2019).

Samples were obtained by masturbation after approximately 48 h of abstinence and subjected to routine analysis following WHO guidelines (World Health Organization, 2010). Only samples that fulfilled WHO normality criteria for semen volume, total sperm number, motility and vitality were included in the study. After liquefaction, samples were divided into two aliquots and motile sperm were selected by the swim-up procedure (World Health Organization, 2010) using NUTR or STRV medium (as stated in each case). Briefly, 0.5–1 ml of semen were placed in a 15-ml sterile conical tube and 1 ml of medium was gently layered over it. Tubes were incubated at 45° angle, at 37°C in a 5% (v/v) CO_2_ atmosphere. After 1 h, the upper layers containing highly motile sperm were recovered and 5 ml of the corresponding medium were added. Samples were centrifuged at 350 × g for 7 min, the supernatants were discarded, fresh media were added and the centrifugation step was repeated in the respective media. The sperm pellets were resuspended in the corresponding medium, sperm concentration was determined with a Neubauer hemocytometer and adjusted to 7 × 10^6^ cells/mL.

When indicated, motile sperm were selected by density gradient centrifugation using Percoll. This product was chosen because it lacks glucose. Isotonic Percoll was prepared with 9 volumes of Percoll and 1 volume of 10x concentrated PBS, supplemented with 0.3% BSA. Eighty and 40% (v/v) Percoll solutions were prepared using NUTR or STRV medium as required. The discontinuous gradients were done in a 15-ml sterile conical tube by layering 1 ml of 40% Percoll over 1 ml of 80% Percoll. Aliquots of 0.5–1 ml of semen were gently placed on top of each gradient and they were centrifuged at 300 × g for 15 min. Sperm pellets were placed in a new tube containing 7 ml of the corresponding medium and samples were centrifuged at 350 × g for 7 min. The washing procedure was repeated using 2 ml of medium. Sperm pellets were resuspended in NUTR or STRV medium and sperm concentration was determined.

To promote capacitation sperm were incubated for the indicated periods of time in 150 µL of the corresponding medium at 37°C in a 5% (v/v) CO_2_ atmosphere. For SER treatment, sperm were first incubated in STRV medium and at the time of recovery, 150 µL of 2x NUTR were added. As a control, sperm incubated in NUTR were diluted at the same time point with 150 µL of NUTR. Replicate experiments were run with different donors, unless indicated.

### Computer-Assisted Sperm Analysis

Sperm motility parameters were evaluated using the Sperm Class Analyzer® system (SCA v.6.2.0.1.; Microptic SL, Barcelona, Spain) that acquires 60 frames per second (s). Sperm suspensions (15 µL) were placed onto a slide, using a 22 × 22 mm^2^ coverslide (preparation depth of 31 µm), and temperature was maintained at constant 37°C using a temperature-controlled stage. At least 5 microscopic fields and at least 300 sperm were analyzed. The following parameters were assessed: curvilinear velocity (VCL, µm/s), straight line velocity (VSL, µm/s), average path velocity (VAP, µm/s), linearity (LIN: VSL/VCL × 100, %), straightness (STR: VSL/VAP × 100, %), wobble (WOB: VAP/VCL × 100, a measure of sperm head side to side movement, %), amplitude of lateral head displacement (ALH, µm, which measures the magnitude of the lateral displacement of the sperm head about the average path) and beat cross frequency (BCF, Hz). Sperm motility was classified as follows: rapid progressive (VCL ≥ 35 μm/s; STR ≥ 80%), medium progressive (VCL ≥ 15 μm/s; STR ≥ 80%), *in situ* (VCL<15 μm/s; VAP ≥ 5 μm/s) and immotile (VAP < 5 μm/s). Percentages of total (rapid progressive + medium progressive + *in situ*) and progressive (rapid + medium progressive) motility were recorded. Drifting was set in 25 μm/s. Sperm were considered hyperactivated when presenting VCL ≥ 150 µm/s, LIN < 50% and ALH ≥ 3.5 µm [modified from (Mortimer et al., 1998)].

### ATP Measurement

ATP levels were determined using a commercial kit (Cat #700410; Cayman Chemical, Ann Arbor, MI, United States). Motile sperm (4 × 10^6^) were incubated for the indicated periods of time under capacitating conditions. The medium was removed by centrifugation (400 × g for 5 min), and the sperm pellet was washed with PBS at room temperature. A second wash was performed with PBS at 4°C, followed by centrifugation at 700 × g for 4 min. Sperm were resuspended in 40 µL of ATP Detection Sample buffer (1x) at 4°C, homogenized by repeated pipetting and stored at −20°C until use. On the day of measurement, samples were thawed, diluted 1:50 with ATP Detection Sample buffer (1x) and maintained on ice. ATP detection standards were prepared and the assay was run following the manufacturer’s instructions. Luminiscence signal was recorded using a GloMax® 96 Microplate Luminometer (Promega; Madison, WI, United States). Results were expressed as pmol/10^6^ sperm and normalized against the control condition.

### Determination of cAMP Levels

Sperm intracellular cAMP levels were determined by radioimmunoassay (RIA). Sperm were centrifuged at 700 × g for 4 min, the supernatant was discarded and 1 ml of PBS at 37°C was added. The centrifugation step was repeated and the sperm pellet was resuspended in 300 μL of absolute ethanol, followed by vortexing for 1 min. The sample was centrifuged at 15,000 × *g* for 4 min, the supernatant was recovered and the ethanol was subjected to evaporation by heating at 55–60°C for 4 h. An aliquot of 220 μL of 50 mM Na-acetate (pH 6.2) was added, it was vortexed for 1 min and the sample was stored at −20°C until cAMP determination as described (Del Punta et al., 1996; Abiuso et al., 2014). cAMP standards of 0–5,000 fmol in 100 μL of Na-acetate buffer were used, and duplicate determinations of the standards and samples (extracts corresponding to 10 × 10^6^ sperm) were done. A 10-μL aliquot of acetic anhydride and triethylamine (1:2 v/v) was added and incubated at room temperature for 10 min. Then, TME-cAMP labeled with I^125^ (25,000 cpm) and the anti-cAMP antibody (1:17,500), both diluted in 100 μL of acetate buffer, were added. After overnight incubation at 4°C, the antigen-antibody complexes were precipitated with 50 μL of 2% BSA and 2 ml of cold ethanol (95%), and samples were centrifuged at 700 × g during 12 min. Supernatant was aspirated and the pellet radioactivity was determined using a Packard Auto-Gamma (Packard Instrument Co., Downers Grove, IL, United States). The inter and intra-assay coefficients of variability were 4.8 and 3.2, respectively. Results were expressed as fmol/10^6^ sperm and normalized against the control condition.

### Determination of Intracellular Ca^2+^ Levels

Sperm [Ca^2+^]_i_ levels were assessed by flow cytometry using Fluo-4 AM. After incubation in the appropriate medium, samples were centrifuged at 400 × g for 5 min at room temperature and resuspended in 250 μL of non-capacitating NUTR or STRV medium (devoid of NaHCO_3_ and BSA), containing 1 μM Fluo-4 AM and 0.02% pluronic acid for 15 min at 37°C in the darkness. Samples were centrifuged again and resuspended in 500 μL of non-capacitating NUTR or STRV medium. Before collecting data, 2 μg/ml of PI was added to monitor viability. When indicated, 100x aliquots of energy sources were added to starved sperm and registered after 1 min. In this case, an equal amount of NUTR medium was added as control. Data were recorded as individual cellular events using a MACSQuant Analyzer 16 cytometer (Miltenyi Biotec Inc.). Forward-scatter area (FSC-A) and side-scatter area (SSC-A) data were collected from 20,000 events per sample in order to define sperm population as previously described (Escoffier et al., 2012). Doublet exclusion was performed analyzing two-dimensional dot plot FSC-A vs. forward-scatter height (FSC-H). Doublets exhibit a higher signal width or area to height ratio compared to single cells (singlets); events deviating from the diagonal are doublets. Positive cells for Fluo-4 AM and PI were collected using the filters for Fluorescein isothiocyanate (FITC; 530/30) and Peridinin chlorophyll protein complex (PerCP; 670LP), respectively. The two indicators had minimal emission overlap, but still compensation was done. Data were analyzed using FlowJo software (V10.0.7). Results were expressed as median fluorescence and normalized against the control condition.

### Protein Extracts, SDS-PAGE and Western Immunoblotting

Motile sperm were incubated in the corresponding medium for the indicated periods of time. Reactions were stopped by adding 500 µL of PBS and cells were centrifuged for 5 min at 600 × g. Sperm pellets were resuspended in Laemmli sample buffer without 2-mercaptoethanol, heated for 5 min at 100°C and centrifuged at 15,000 × g for 5 min. The supernatants were recovered and stored at −20°C until used. Samples were supplemented with 5% 2-mercaptoethanol, boiled for 5 min, and subjected to SDS-PAGE in 10% polyacrylamide gels and Western immunoblotting. To analyze pPKAs, membranes were developed with anti-pPKAs (1:3,000) and anti-rabbit IgG conjugated with HRP. To evaluate pY, primary antibody (1:5,000) and anti-mouse conjugated with HRP were used. As loading control, membranes were developed with anti-β-tubulin (1:5,000). The reactive bands were detected by enhanced chemiluminiscence (ECL) using standard procedures. The same membranes were developed with each of the 3 primary antibodies, followed by stripping with 0.2 N NaOH for 2 min. In some cases, pPKAs was first developed; in other cases, pY was detected in the first place. Western immunoblot images were analyzed with ImageJ 1.48 k (National Institute of Health, United States), following the specifications of ImageJ User Guide, IJ 1.46r. The optical densities of all bands, from 250 to 25 kDa, were quantified and expressed as relative to the β-tubulin band. Results from the NUTR condition were considered 100%.

### Statistical Analysis

Statistical analyses were done using the GraphPad Prism program (version 6.01 for Windows; GraphPad Software, San Diego, CA, United States). Data were expressed as mean ± standard error of the mean (SEM). To assume normal distribution, percentages were converted to ratios and subjected to the arcsine square root transformation. Results were compared by one or two-way analysis of variance (ANOVA) and multiple comparison tests, or Student’s t test, as indicated. A *p* value of < 0.05 was considered statistically significant.

## Results

### Human Sperm Remain Motile but do Not Undergo Hyperactivation in the Absence of Exogenous Nutrients

**FIGURE 1:**
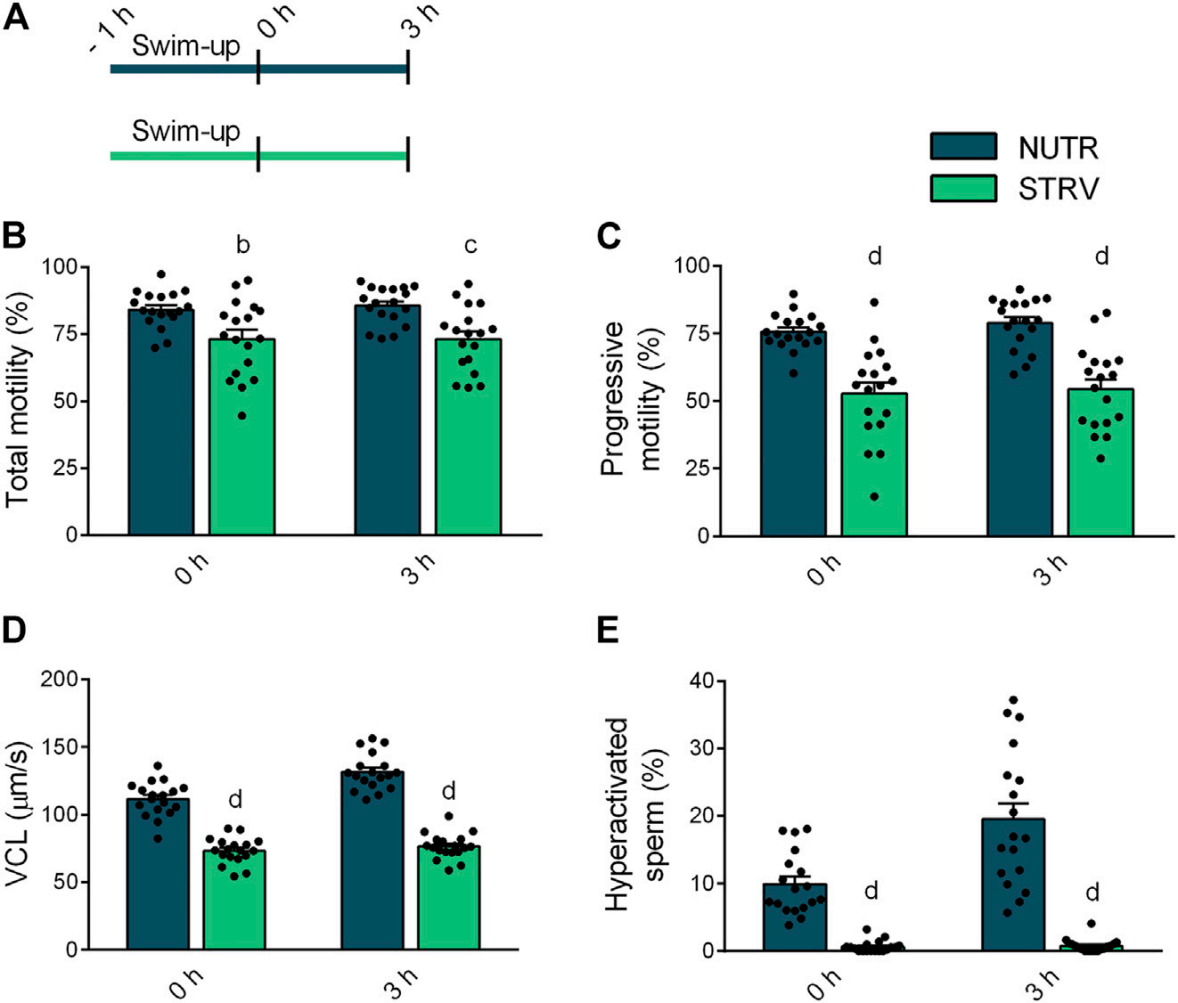
Sperm motility in the presence or absence of exogenous nutrients. **(A)** Experimental design. Motile human sperm were selected by swim-up in BWW containing glucose, lactate and pyruvate (NUTR) or in medium devoid of glucose, pyruvate and lactate (STRV). Motility parameters were recorded by CASA immediately after swim-up (0 h) and after 3-h incubation in the same medium in capacitating conditions. **(B)** Total motility. **(C)** Progressive motility. **(D)** Curvilinear velocity (VCL). **(E)** Hyperactivated sperm. Results are expressed as mean ± SEM, *n* = 18 experiments, 8 donors (≤3 samples per donor). ^b^ *p* < 0.01, ^c^ *p* < 0.001, ^d^ *p* < 0.0001 vs. NUTR. Two-way ANOVA, and Bonferroni’s multiple comparison test.

To determine the effect of starvation on human sperm motility, we split each semen sample into two aliquots and performed the swim-up procedure using BWW lacking glucose, pyruvate and lactate (STRV) or medium with nutrients (NUTR), as control. After swim-up, motile sperm were washed twice and resuspended in STRV or NUTR media, respectively. Motility parameters were then evaluated by CASA either immediately after the swim-up (0 h) or after 3-h incubation (Figure 1A). At both time points, sperm incubated in NUTR medium showed high percentages of total and progressive motility (Figures 1B,C). However, contrary to our observations with mouse sperm (Navarrete et al., 2019), human sperm continued to be motile when incubated for 3 h in the STRV condition (Figure 1B). In the absence of exogenous substrates, the population of sperm showing progressive motility was significantly reduced (Figure 1C) and the average VCL was diminished compared to those of the NUTR medium (Figure 1D). In addition to VCL, sperm incubated in the STRV condition also displayed lower average values on VSL, VAP, WOB, ALH and BCF (Table 1). Moreover, in the absence of energy metabolites, sperm did not undergo hyperactivation (Figure 1E). Similar results were obtained when motile sperm were selected by density gradient centrifugation using Percoll (Supplementary Figure S1 and Supplementary Table S1).

**TABLE 1:** Kinematic characteristics of human sperm evaluated after swim-up (0 h) and incubated for 3 h in medium with or without nutrients (NUTR and STRV, respectively). Results are expressed as mean ± SEM, *n* = 18 experiments, 8 donors (≤3 samples per donor).^b^ *p* < 0.01,^d^ *p* < 0.0001 vs. NUTR. Two-way ANOVA, and Bonferroni’s multiple comparison test.

	NUTR 0 h	STRV 0 h	NUTR 3 h	STRV 3 h
VCL (μm/s)	112 ± 3	73 ± 2^d^	132 ± 3	77 ± 2^d^
VSL (μm/s)	39 ± 2	26 ± 2^d^	41 ± 3	25 ± 2^d^
VAP (μm/s)	64 ± 2	45 ± 1^d^	72 ± 2	46 ± 2^d^
LIN (%)	36 ± 2	36 ± 2	33 ± 3	33 ± 2
STR (%)	59 ± 2	55 ± 3	56 ± 3	53 ± 2
WOB (%)	58 ± 1	62 ± 1 ^b^	55 ± 2	60 ± 1 ^b^
ALH (μm)	2.5 ± 0.1	1.7 ± 0.1^d^	2.9 ± 0.2	1.8 ± 0.1^d^
BCF (Hz)	16 ± 1	12 ± 1^d^	16 ± 1	12 ± 1^d^

**FIGURE 2:**
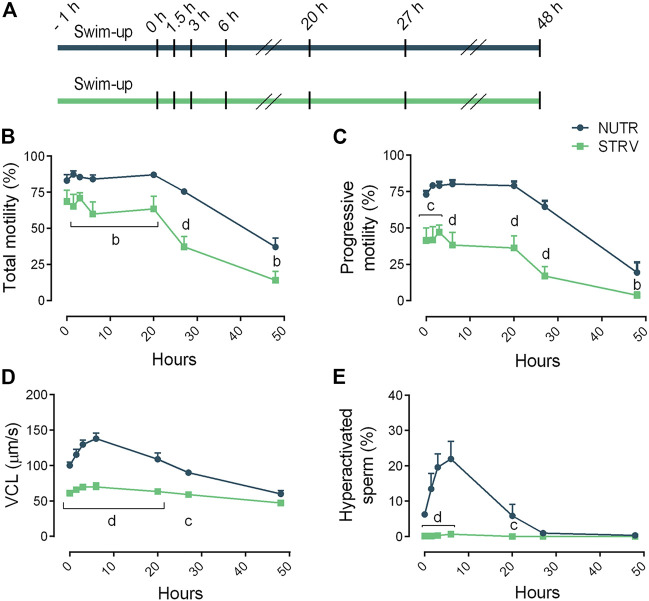
Sperm motility after incubation for long periods of time in the presence or absence of exogenous nutrients. **(A)** Experimental design. Motile human sperm were selected and incubated in capacitating conditions in BWW containing glucose, lactate and pyruvate (NUTR) or in medium devoid of glucose, pyruvate and lactate (STRV). Motility parameters were recorded by CASA at 0, 1.5, 3, 6, 20, 27 and 48 h. **(B)** Total motility. **(C)** Progressive motility. **(D)** Curvilinear velocity (VCL). **(E)** Hyperactivated sperm. Results are expressed as mean ± SEM, *n* = 5 experiments. ^b^ *p* < 0.01, ^c^ *p* < 0.001, ^d^ *p* < 0.0001 for STRV vs. NUTR at each corresponding time. Two-way ANOVA, and Bonferroni’s multiple comparison test.

Overall, these data indicate that, contrary to mouse sperm, human sperm remain motile in media devoid of energy nutrients suggesting that human sperm can obtain sufficient energy to continue moving, possibly from internal sources. To further evaluate this possibility, in another set of experiments, sperm were incubated for up to 48 h in STRV or NUTR media and motility parameters were evaluated at different time points (Figure 2A). As expected, in both conditions there was a decline in total motility with increasing incubation times (Figure 2B). However, even after 48-h incubation, a fraction of the sperm population continued to move despite the absence of exogenous metabolites. Other parameters such as the percentage of progressive motility also decreased with time (Figure 2C). Noteworthy, VCL was maintained in the STRV condition during the entire period of the experiment (Figure 2D) and no hyperactivation was observed in these cells at any of the time points analyzed (Figure 2E). Significantly reduced values were obtained for most of the kinematic parameters evaluated when sperm were incubated in STRV (Supplementary Figure S2).

### OXPHOS Is Involved in the Maintenance of Human Sperm Motility in Starving Condition

**FIGURE 3:**
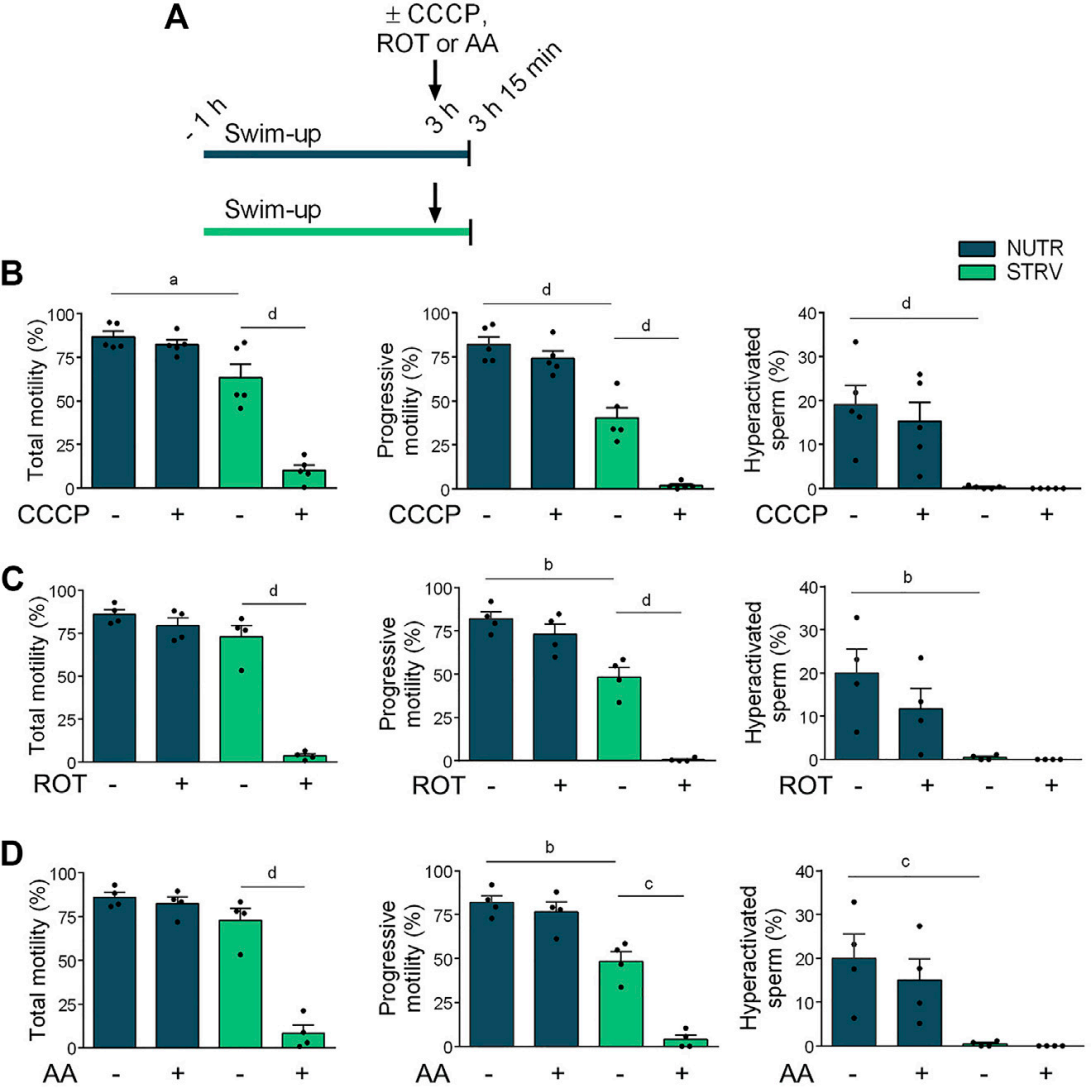
Effect of incubation with OXPHOS inhibitors on human sperm motility. **(A)** Experimental design. Motile human sperm were selected and incubated for 3 h in NUTR or STRV medium. The OXPHOS inhibitors (10 µM CCCP, 1 µM ROT or 0.3 µM AA) were added (indicated with arrows) and after 15 min, motility was recorded by CASA. **(B)** Motility parameters in sperm exposed to CCCP. **(C)** Motility parameters in sperm exposed to ROT. **(D)** Motility parameters in sperm exposed to AA. Results are expressed as mean ± SEM, *n* ≥ 4 experiments. ^a^ *p* < 0.05, ^b^ *p* < 0.01, ^c^ *p* < 0.001, ^d^ *p* < 0.0001. One-way ANOVA, and Tukey’s multiple comparison test.

To determine the contribution of OXPHOS in the maintenance of human sperm motility in the absence of energy nutrients, sperm incubated either in NUTR or STRV medium were exposed the last 15 min to carbonyl cyanide *p*-chlorophenylhydrazone (CCCP, a mitochondrial uncoupler), rotenone (ROT, an inhibitor of complex I of mitochondrial electron transport chain) or antimycin A (AA, an inhibitor of complex III of mitochondrial electron transport chain) and motility parameters were assessed (Figure 3A). In NUTR medium, no significant decline in any of the motility parameters was observed when sperm were exposed to either CCCP, ROT or AA (Figures 3B–D). However, cells incubated in starvation stopped moving when OXPHOS was inhibited (Figures 3B–D). These results indicate that in nutrients containing medium, glycolysis is sufficient to maintain sperm motility and hyperativation. However, in the absence of exogenous energy substrates, sperm motility is maintained exclusively by OXPHOS-mediated ATP production.

### Human Sperm Motility and Hyperactivation Are Recovered After SER Treatment

**FIGURE 4:**
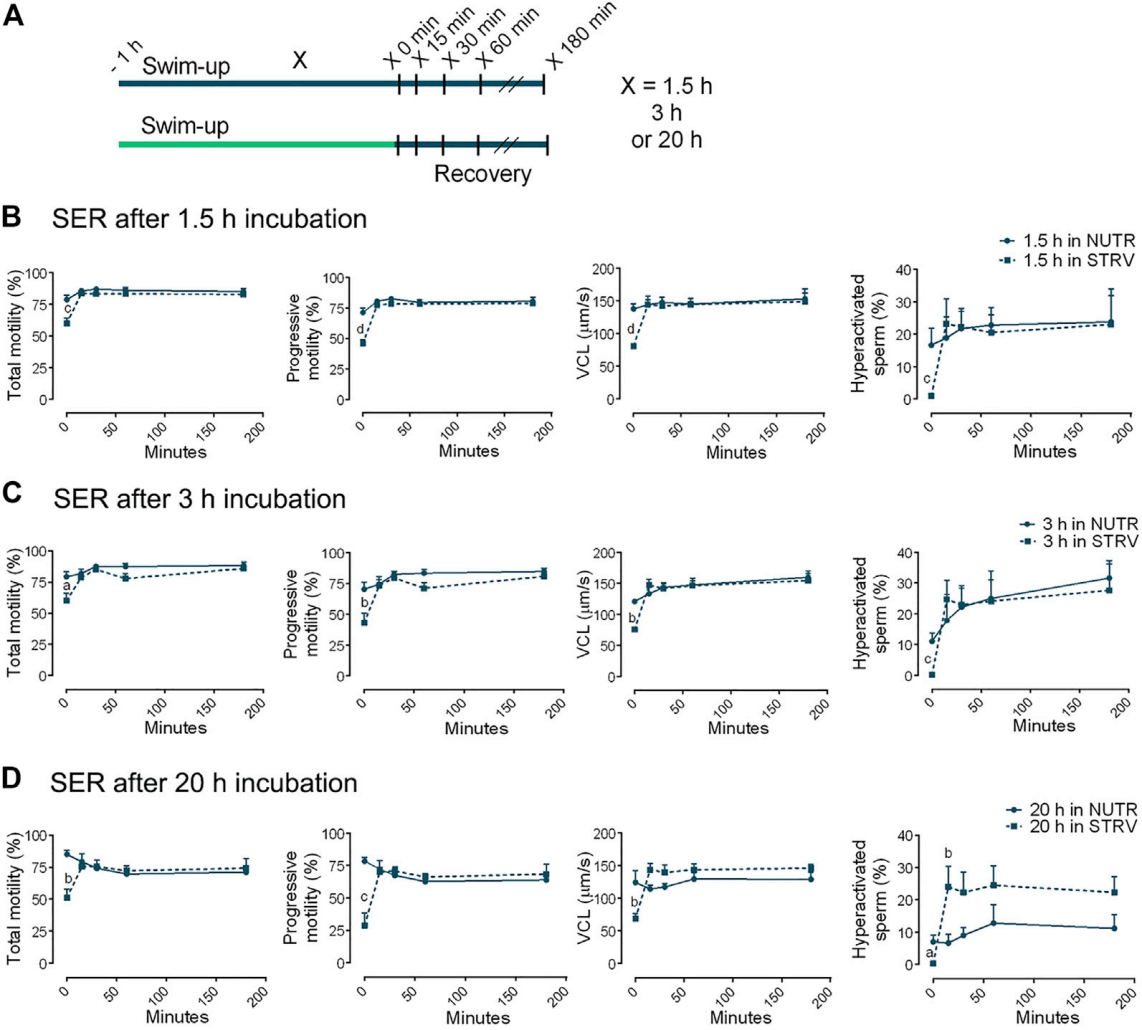
Sperm energy restriction and recovery (SER) treatment after incubation for different periods of time. **(A)** Experimental design. Motile human sperm were selected and incubated in capacitating conditions in STRV medium for 1.5, 3 or 20 h. The energy recovery was done by adding the same volume of 2x NUTR. As a control, sperm were incubated for 1.5, 3 or 20 h in NUTR medium, and the same volume of NUTR medium was added at the time of recovery. Motility parameters were recorded by CASA before (time 0 min) and after 15, 30, 60 and 180 min of energy recovery. **(B)** Parameters for SER treatment after 1.5-h incubation in NUTR (full line) and STRV (dotted line). **(C)** Parameters for SER treatment after 3-h incubation in NUTR (full line) and STRV (dotted line). **(D)** Parameters for SER treatment after 20-h incubation in NUTR (full line) and STRV (dotted line). Results are expressed as mean ± SEM, *n* = 4 experiments. ^a^ *p* < 0.05, ^b^ *p* < 0.01, ^c^ *p* < 0.001, ^d^ *p* < 0.001 for STRV vs. NUTR at each corresponding time. Two-way ANOVA, and Bonferroni’s multiple comparison test.

Based on the evidence in the murine and bovine models (Navarrete et al., 2019), we aimed to determine the effect of SER treatment on human sperm motility. After swim-up, sperm were incubated for different periods of time (1.5, 3 or 20 h) in STRV medium. Then, energy substrates were added, and motility was recorded at 15, 30, 45, 60 and 180 min (Figure 4A). Sperm incubated for 1.5 and 3 h under energy restriction were able to recover all motility parameters after addition of energy substrates, at least to similar levels than those consistently incubated in the presence of nutrients (Figures 4B,C). Interestingly, addition of exogenous nutrients to sperm starved for 20 h not only allowed the recovery of total and progressive motility and VCL, but also led to significantly higher hyperactivation percentages in comparison to the control (Figure 4D). The hyperactivation values obtained in SER treatment after 20-h incubation were similar to those of cells incubated for 3 h in NUTR medium (Figure 4C).

### Capacitation-Associated Events Are Differentially Regulated During Sperm Starvation and Recovery

**FIGURE 5:**
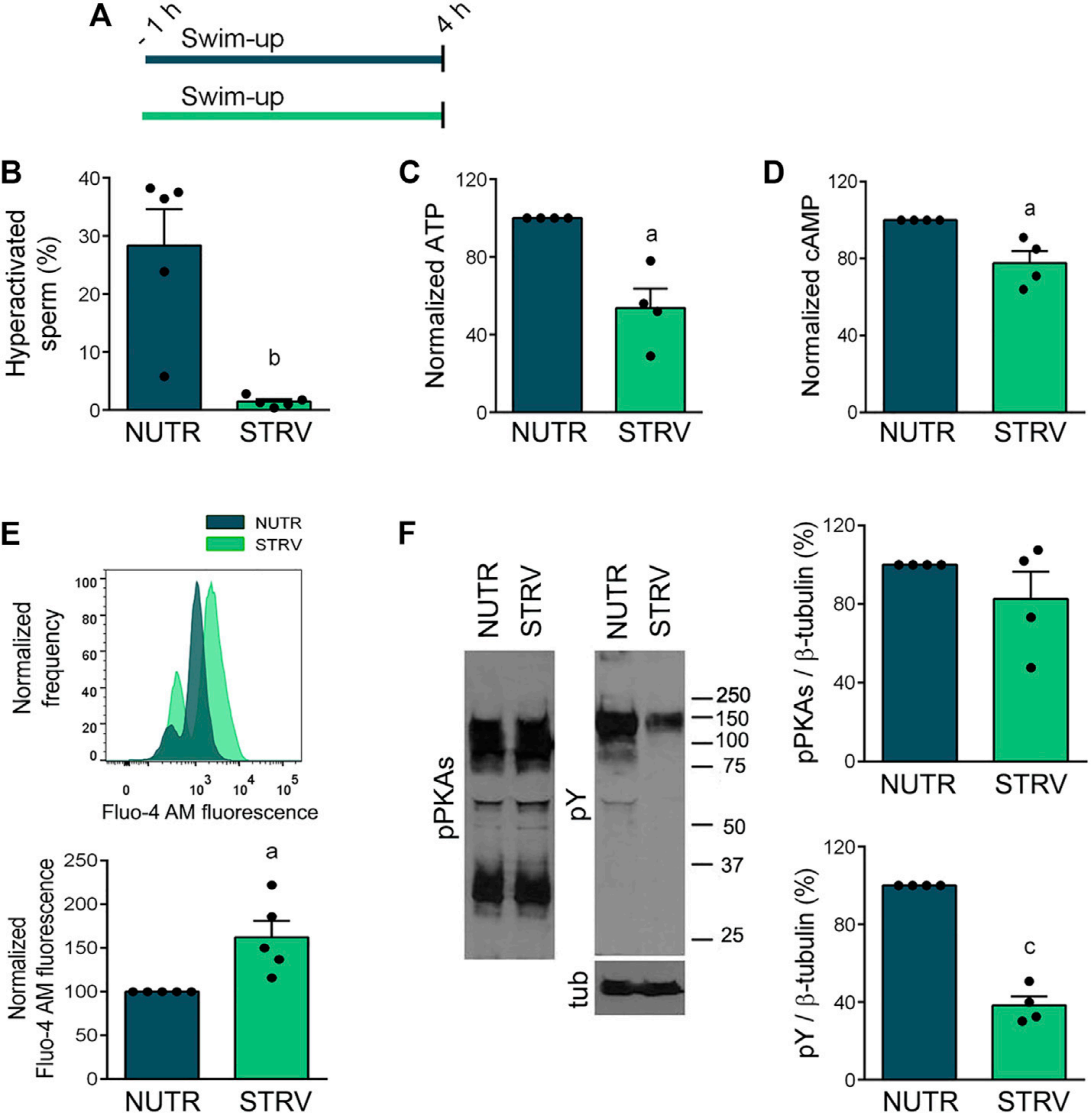
Capacitation-related events in sperm incubated in starving condition. **(A)** Experimental design. Motile human sperm were selected by swim-up and incubated for 4 h in capacitating conditions in NUTR or STRV media. **(B)** Hyperactivated sperm. **(C)** ATP levels normalized against NUTR condition. **(D)** cAMP levels normalized against NUTR condition. **(E)** [Ca^2+^]_i_ levels. Top: Representative histograms of normalized frequency vs. Fluo-4 AM fluorescence of non-PI stained sperm (live) are shown. Bottom: Normalized Fluo-4 AM fluorescence compared to NUTR condition. **(F)** pPKAs and pY levels. Left: Representative Western immunoblotting results are shown. Right: Protein signals were quantified and expressed as relative to β-tubulin (Tub). Results are expressed as mean ± SEM, *n* ≥ 4 experiments. ^a^ *p* < 0.05, ^b^ *p* < 0.01, ^c^ *p* < 0.001 vs. NUTR. Student’s t test.

Sperm capacitation is associated with the acquisition of hyperactivated motility, which has high ATP demands (Edwards et al., 2007; Hereng et al., 2011). Moreover, during capacitation there is an activation of the cAMP/PKA pathway that leads to increased phosphorylation of PKA substrates (pPKAs) and tyrosine residues (pY) (Gervasi and Visconti, 2016; Puga Molina et al., 2018). First, we analyzed the effect of starvation on several capacitation-associated parameters (Figure 5A). As observed in previous experiments, sperm incubated for 4 h under starvation did not develop hyperactivated motility (Figure 5B and Supplementary Figure S3). These cells showed lower levels of ATP and cAMP in comparison to sperm incubated in the presence of energy substrates (Figures 5C,D). Similar to our findings with mouse sperm (Sánchez-Cárdenas et al., 2021), starvation increased [Ca^2+^]_i_ (Figure 5E). Regarding protein phosphorylation, while no significant differences were obtained for pPKAs, pY was significantly reduced in these cells (Figure 5F).

**FIGURE 6:**
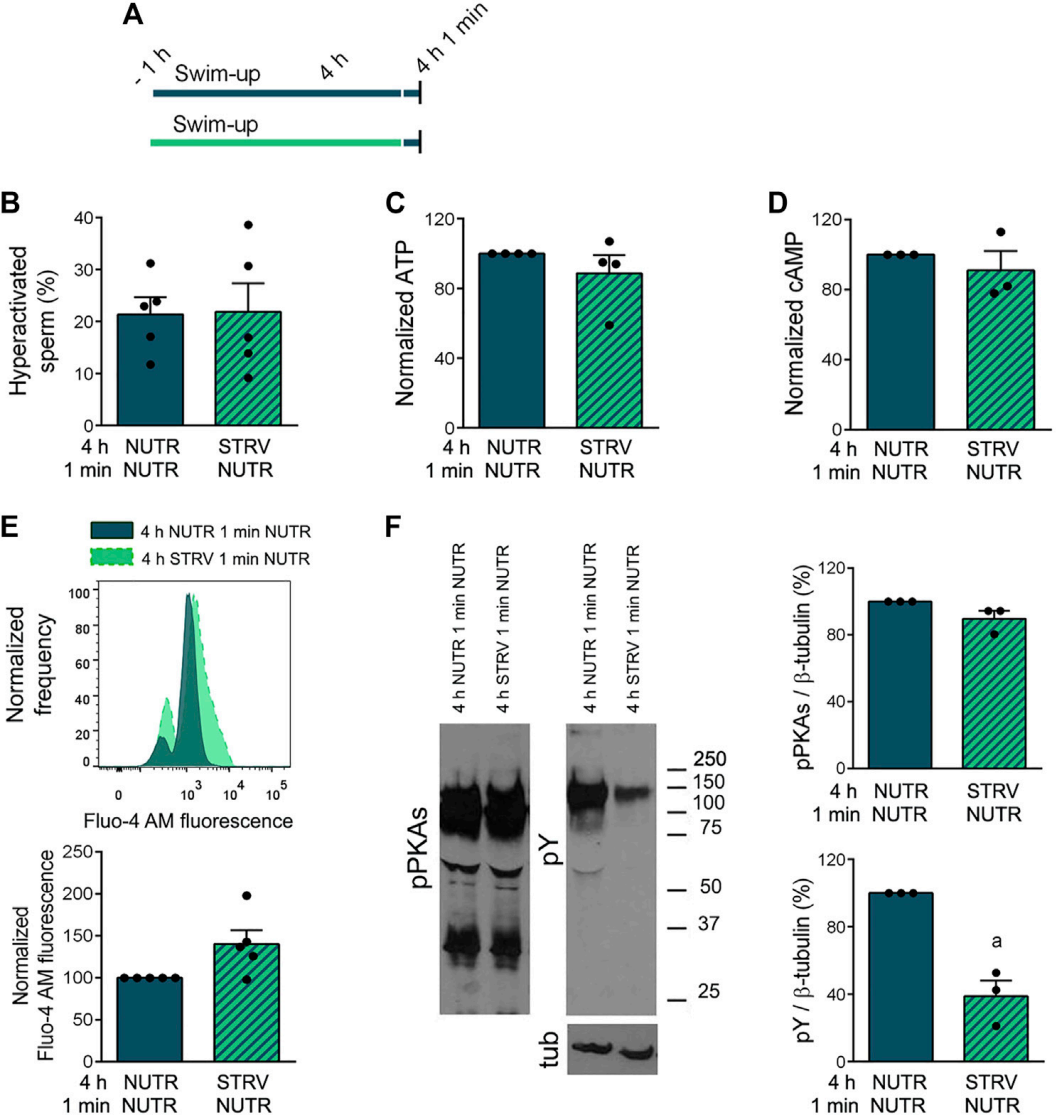
Capacitation-related events in sperm incubated in starving condition and subjected to recovery in NUTR medium for 1 min. **(A)** Experimental design. Motile human sperm were selected by swim-up and incubated for 4 h in capacitating conditions in NUTR or STRV, and then exposed to NUTR medium for 1 min. **(B)** Hyperactivated sperm. **(C)** ATP levels normalized against 4 h NUTR 1 min NUTR condition. **(D)** cAMP levels normalized against 4 h NUTR 1 min NUTR condition. **(E)** [Ca^2+^]_i_ levels. Top: Representative histograms of normalized frequency vs. Fluo-4 AM fluorescence of non-PI stained sperm (live) are shown. Bottom: Normalized Fluo-4 AM fluorescence compared to 4 h NUTR 1 min NUTR condition. **(F)** pPKAs and pY levels. Left: Representative Western immunoblotting results are shown. Right: Protein signals were quantified and expressed as relative to β-tubulin (Tub). Results are expressed as mean ± SEM, *n* ≥ 3 experiments. ^a^ *p* < 0.05 vs. 4 h NUTR 1 min NUTR. Student’s t test.

Next, starved sperm were subjected to recovery in NUTR medium for 1 min and sperm parameters were analyzed (Figure 6A). One-min incubation in the presence of exogenous nutrients led to the development of sperm hyperactivation (Figure 6B and Supplementary Figure S3). The 1-min recovery treatment also resulted in an increase in both ATP and cAMP concentrations (Figures 6C,D) and a decrease in [Ca^2+^]i (Figure 6E). Under these conditions, no improvement in pY levels were observed (Figure 6F).

## Discussion

The ionic requirements and signaling pathways of mammalian sperm motility and capacitation have been widely studied, but they are not completely unraveled (Puga Molina et al., 2018; Vyklicka and Lishko, 2020). Moreover, the energy demands of sperm motility and the metabolic changes associated to mammalian sperm capacitation are scarcely known. Our recent findings in the mouse indicate that capacitation is associated with increased glucose and oxygen consumption, with the involvement of cAMP and Ca^2+^ signaling pathways (Balbach et al., 2020; Hidalgo et al., 2020). To further study changes in metabolism during this process, we developed the SER methodology in which mouse and bovine sperm are transiently starved until they stop moving and then are recovered by addition of energy substrates (Navarrete et al., 2019). The promising results in terms of reproductive performance obtained in these animal models prompted us to analyze the effect of starvation and recovery on human sperm function.

In the present study, we found that contrary to mouse sperm, human sperm can be incubated in the absence of energy nutrients while maintaining a significant percentage of motile cells for several hours. In our work, human sperm were first incubated for 3 h in medium devoid of glucose, pyruvate and lactate, and motility parameters were analyzed. Under these conditions, there was an overall decline in the percentages of total and progressive motility, and in most kinematic parameters. However, the decline was moderate and over 50% of the sperm population continued moving with kinematic parameters reduced to about 70% of those found when consistently exposed to energy nutrients. Interestingly, it was previously shown that sperm incubated at 22 or 4°C (but not at 37°C) can survive and remain motile for several days when incubated in PBS with or without addition of glucose (Amaral et al., 2011). It is worth mentioning that human semen contains high levels of fructose (Owen, 2005), so it was possible that remaining glycolysable substrates fueled glycolysis. To minimize seminal plasma contamination, cells were washed twice after the swim-up procedure and motile sperm were also selected by density gradient centrifugation using Percoll. However, after both treatments, human sperm incubated in the absence of exogenous nutrients do not stop moving suggesting that fructose traces would not be responsible of maintaining motility. The differences in the response between human and mouse sperm to starvation can be attributed to intrinsic metabolic variations in sperm from these species. Alternatively, it is important to consider that while human sperm are ejaculated cells, mouse sperm are recovered from cauda epididymides, and that contact with seminal plasma might alter sperm metabolism (Pérez-Patino et al., 2019).

To further analyze human sperm motility in starvation, sperm were incubated under this condition for up to 48 h. Surprisingly, high percentages of total and progressive motility were maintained for more than 27 h, but no hyperactivation was observed at any time point analyzed. Our results also showed that starved sperm stop moving when exposed to CCCP, ROT or AA, indicating that in the absence of exogenous sources, ATP is produced by OXPHOS. Altogether, these results suggest the existence of alternative metabolic pathways involving endogenous substrates that maintain energy supplies in human sperm subjected to starvation.

The relevance of glycolysable and non-glycolysable substrates to support hyperactivation and capacitation has been previously reported for sperm of several mammalian species (Fraser and Herod, 1990; Rigau et al., 2002; Mukai and Okuno, 2004; Goodson et al., 2012; Balbach et al., 2020). Regarding human sperm, the presence of glucose has been reported to be essential to support capacitation-related events (Rogers and Perreault, 1990; Williams and Ford, 2001; Amaral et al., 2011; Carrageta et al., 2020). However, the source of ATP and the contribution of glycolysis and OXPHOS to maintain human sperm function remain uncertain (Nascimento et al., 2008; Piomboni et al., 2012). A recent study was focused on the identification of endogenous metabolites in human sperm cells (Paiva et al., 2015). Interestingly, they found that the overrepresented pathways included not only the metabolism of carbohydrates, but also of lipids and proteins (Paiva et al., 2015). Moreover, nearly 25% of the proteins found in a proteome of human sperm flagellum are involved in lipid metabolism, and oxidation of fatty acids has been suggested as human sperm ATP source in the absence of exogenous substrates (Amaral et al., 2013). Another possible source of sperm energy in the absence of exogenous glycolysable substrates lies in glycogen stores and/or gluconeogenesis activation. Granules of glycogen have been described in sperm from some invertebrate and vertebrate species (Anderson and Personne, 1970; Michalik et al., 2005; Luo et al., 2011; Miquel et al., 2013). The presence of glycogen has been shown in dog sperm (Palomo et al., 2003; Albarracín et al., 2004) and some gluconeogenesis metabolites have been identified in human sperm (Paiva et al., 2015). Although in the seminal plasma and in the female tract, sperm are exposed to exogenous nutrients, more work is needed to understand the extent by which endogenous energy sources have a role in maintaining sperm function.

Our results showed that human sperm incubated in starvation displayed lower ATP and cAMP levels than cells capacitated in standard medium. Such levels were sufficient to promote the phosphorylation of PKA substrates, but did not support phosphorylation on tyrosine residues or hyperactivated motility. Regarding the phosphorylation of PKA substrates, when sperm are exposed to seminal plasma there is an early HCO_3_^−^-dependent activation of soluble adenylyl cyclase (sAC) (Okamura et al., 1985), which in turn leads to cAMP synthesis and PKA activation (Buffone et al., 2014). It is possible that in our experimental conditions, this early activation of sAC is sufficient to produce enough levels of cAMP, together with the fact that sperm still display significant amounts of ATP to be used by sAC. However, after 1-min exposure to the exogenous substrates, starved sperm were able to develop hyperactivated motility. Such response was accompanied by an increase in ATP and cAMP levels, with no changes in pPKAs or pY levels. These results indicate that occurrence of pY is dispensable for achieving hyperactivated motility. In this regard, we recently reported that mice carrying an inactive tyrosine kinase FER do not undergo capacitation-related pY but are fertile *in vivo* (Alvau et al., 2016), suggesting that pY is not essential for mouse sperm fertility or that its absence can be compensated by factors/mechanisms inherent of the female reproductive tract.

The development of hyperactivated motility is highly influenced by ion fluxes, in particular, by intracellular levels of Ca^2+^ brought in by CatSper channels (Vyklicka and Lishko, 2020). The almost immediate recovery of hyperactivation upon rescue (SER treatment) may be indicative that critical ion fluxes are rapidly restored. Although there is no evidence that CatSper is sensitive to ATP, other transporters or pumps are ATP-driven. For example, the Na^+^, K^+^-ATPase α4 isoform, the plasma membrane Ca^2+^/calmodulin dependent ATPase, isoform 4 (PMCA4) or the sarcoplasmic/endoplasmic reticulum Ca^2+^ ATPases (SERCAs) (Blanco and Mercer, 1998; Withers et al., 2006; Lawson et al., 2007). Consistently, we have recently shown in mouse sperm that in the absence of exogenous energy nutrients, there is an increase in [Ca^2+^]_i_ (Sánchez-Cárdenas et al., 2021). However, such increase in [Ca^2+^]_i_ was also observed in sperm from *CatSper1* null mice suggesting the involvement of other Ca^2+^ transporters. In agreement with these findings, starved human sperm displayed increased [Ca^2+^]_i_ compared to cells incubated with nutrients, and there was a decrease in Ca^2+^ levels after the 1-min recovery. The inverse relationship between [Ca^2+^]_i_ and pY levels was observed in previous studies under different experimental conditions (Marín-Briggiler et al., 2003; Marín-Briggiler et al., 2005; Li et al., 2014) and indicate that the decrease in [Ca^2+^]_i_ obtained in SER-treated sperm would be accompanied by an increase in pY over time.

**FIGURE 7:**
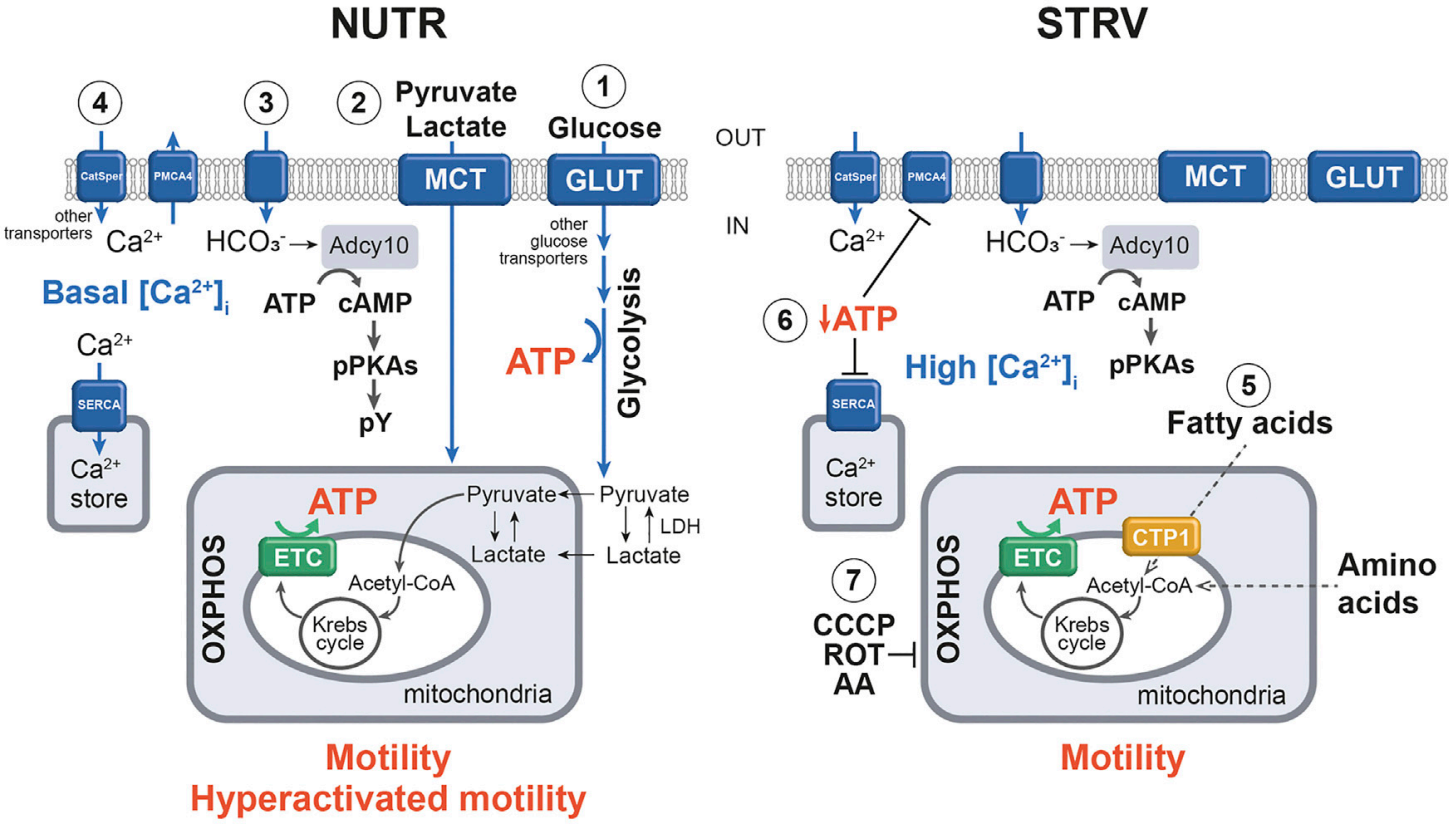
Simplified model of the molecular mechanisms underlying sperm incubation in the presence and absence of exogenous nutrients. 1) In NUTR condition, glucose is transported into sperm through GLUT and other active transporters, and enters the glycolytic pathway, producing ATP. The final product of glycolysis is pyruvate, which can be converted to lactate by LDH. 2) Exogenous pyruvate and lactate can also enter sperm plasma membrane by MCT and be transported to the mitochondria for further metabolization by the Krebs cycle and OXPHOS, which also generate ATP. 3) Influx of HCO_3_^−^ stimulates Adcy10 with the production of cAMP and activation of PKA, which in turn, provokes the phosphorylation of substrates. 4) Under this condition, Ca^2+^ enters sperm mainly through CatSper channels and [Ca^2+^]_i_ levels are regulated by ATP-driven pumps (PMCA4 and SERCA). As a result, sperm can maintain motility and develop hyperactivation. 5) In the absence of exogenous substrates, ATP would be produced by fatty acid oxidation and/or by amino acid metabolism. Such ATP allows the production of sufficient levels of cAMP to stimulate pPKAs. 6) Decreased ATP concentrations inhibit the normal operation of Ca^2+^ pumps and transporters, increasing the [Ca^2+^]_i_. All these events allow the development of motility but not hyperactivation. 7) In STRV, the addition of CCCP, ROT and AA blocks OXPHOS, resulting in motility loss. To simplify this model, transporters and some enzymes are named by generic terms. Filled arrows indicate activation; dotted arrows show hypothetic pathways. NUTR, medium supplemented with glucose, pyruvate and lactate; GLUT, glucose transporter; LDH, lactate dehydrogenase; MCT, monocarboxylate transporter; PMCA4, plasma membrane Calcium ATPase 4; SERCA, sarcoplasmic/endoplasmic reticulum Ca^2+^ ATPases; Acetyl-CoA, acetyl coenzyme A; OXPHOS, oxidative phosphorylation; ETC, mitochondrial electron transport chain; Adcy10, atypical soluble adenylyl cyclase; PKA, protein kinase A; pPKAs, phosphorylation in PKA substrates; pY, phosphorylation on tyrosine residues; STRV, starving conditions; CTP1, carnitine palmitoyl transferase I; CCCP, carbonyl cyanide *p*-chlorophenylhydrazone (a mitochondrial uncoupler); ROT, rotenone (inhibitor of ETC complex I); AA, antimycin A (inhibitor of ETC complex III).

In conclusion, when human sperm are incubated in the absence of exogenous nutrients, motility and kinematic parameters are significantly reduced; however, they are capable to continue moving for several hours. Figure 7 summarizes our findings in a proposed model. Our results also show that motility of starved sperm can be fully recovered upon addition of energy sources. Interestingly, for those sperm incubated in starvation for 20 h, addition of nutrients increase the percentages of hyperactivated cells over those obtained in control conditions. Such results are important from a clinical point of view and would be used to regulate sperm fertilization competence *in vitro*. In mouse and bovine sperm, the most relevant observations were related to post-fertilization events. In both species, the SER treatment improved embryo development rates. In the mouse model, blastocysts derived from zygotes obtained with starved sperm gave significantly more pups than those from control zygotes. Importantly, the SER treatment also improved intracytoplasmic sperm injection (ICSI) results when used in bovines. In this specie, zygotes obtained using ICSI had very low two-cell embryos formation (cleavage) rates and they did not arrive to blastocysts. On the other hand, when SER-treated bull sperm were injected into cow oocytes, we observed over 5 times increased cleavage rates and 17% of two-cell embryos arrived to blastocysts (Navarrete et al., 2019). The results obtained in these animal models, using both IVF and ICSI protocols, prompted us to speculate the possible use of the SER treatment to improve assisted reproductive techniques in humans. Further studies in the clinical setting will be necessary to determine the extent by which human sperm starvation would enhance embryo development and pregnancy rates after IVF and/or ICSI procedures.

## Data Availability

The raw data supporting the conclusion of this article will be made available by the authors, without undue reservation.

## Supplementary Material

Supplementary Figure S1 Sperm motility in the presence or absence of exogenous nutrients. **(A)** Experimental design. Motile human sperm were selected by density gradient centrifugation using Percoll and medium containing glucose, lactate and pyruvate (NUTR) or devoid of glucose, pyruvate and lactate (STRV). Motility parameters were recorded by CASA immediately after selection (0 h) and after 3-h incubation in the same medium in capacitating conditions. **(B)** Total motility. **(C)** Progressive motility. **(D)** Curvilinear velocity (VCL). **(E)** Hyperactivated sperm. Results are expressed as mean ± SEM, *n* = 3 experiments. a *p* < 0.05 vs. NUTR. Two-way ANOVA, and Bonferroni’s multiple comparison test.

Supplementary Figure S2 Kinematic characteristics of human sperm incubated for long periods of time in the presence or absence of exogenous nutrients. Motile human sperm were selected and incubated in capacitating conditions in BWW containing glucose, lactate and pyruvate (NUTR) or in medium devoid of glucose, pyruvate and lactate (STRV). Motility parameters were recorded by CASA at 0, 1.5, 3, 6, 20, 27 and 48 h. Straight line velocity (VSL), average path velocity (VAP), linearity (LIN), straightness (STR), wobble (WOB), amplitude of lateral head displacement (ALH) and beat cross frequency (BCF) were determined. Results are expressed as mean ± SEM, *n* = 5 experiments. ^a^ *p* < 0.05, ^b^ *p* < 0.01, ^c^ *p* < 0.001, ^d^ *p* < 0.0001 for STRV vs. NUTR at each corresponding time. Two-way ANOVA, and Bonferroni’s multiple comparison test.

Supplementary Figure S3 Trajectories of human sperm incubated in the presence or absence of exogenous nutrients. **(A)** Sperm incubated for 4 h in NUTR medium. **(B)** Sperm incubated for 4 h in STRV medium. **(C)** Sperm incubated for 4 h in NUTR and then exposed to NUTR medium for 1 min. **(D)** Sperm incubated for 4 h in STRV and then exposed to NUTR medium for 1 min. Trajectories were obtained by the CASA system, VCL is shown in red, VSL in blue and VAP in green. Immotile cells are shown as yellow circles.
