# Supplementary figures and images for: Human Sperm Remain Motile After a Temporary Energy Restriction but do Not Undergo Capacitation-Related Events
Source: Front Cell Dev Biol. 2021 Nov 12;9:777086. doi: 10.3389/fcell.2021.777086 (PMC8633110; doi:10.3389/fcell.2021.777086)

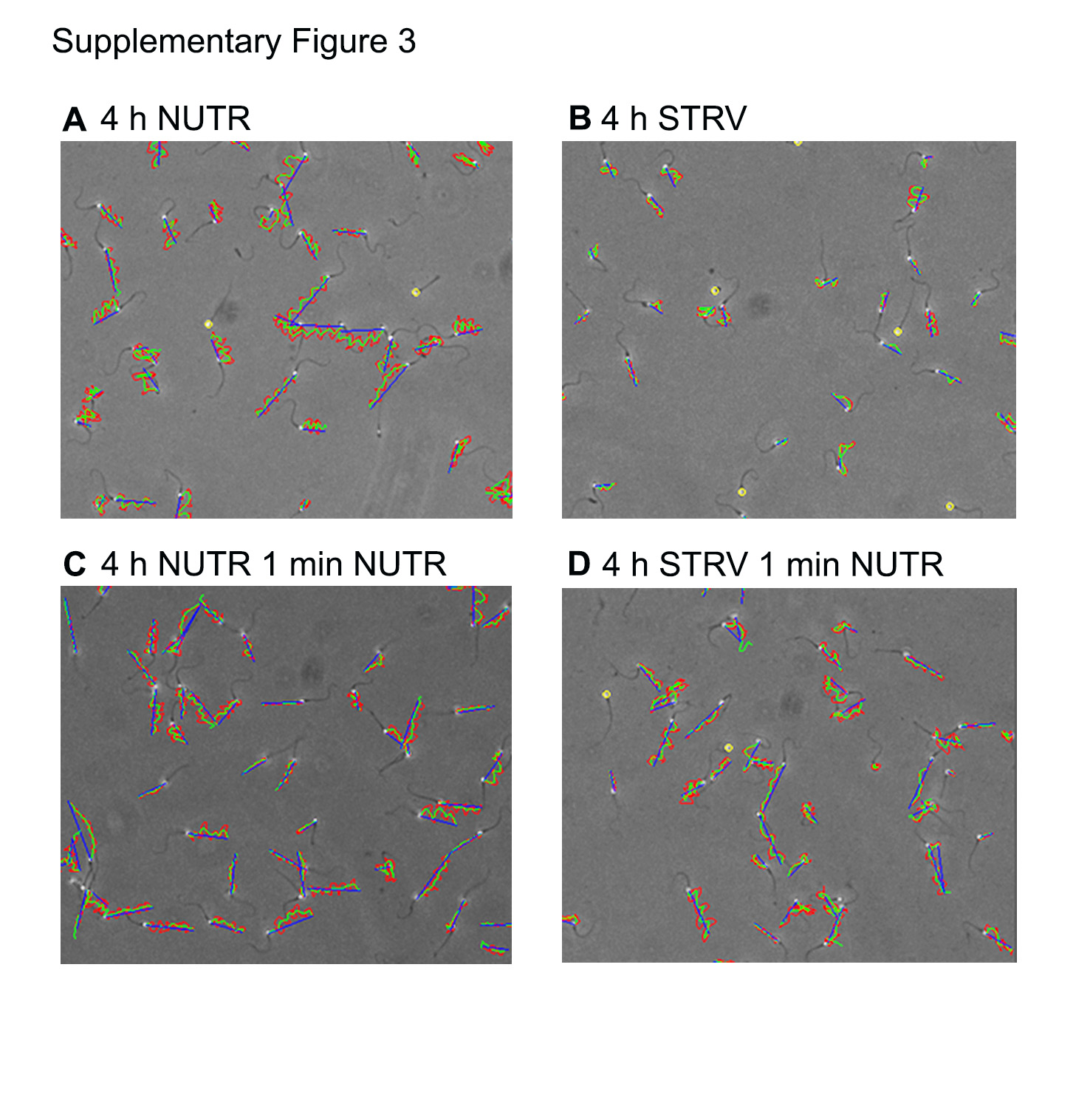

Supplement: Supplementary file 1 [file Image3.JPEG]

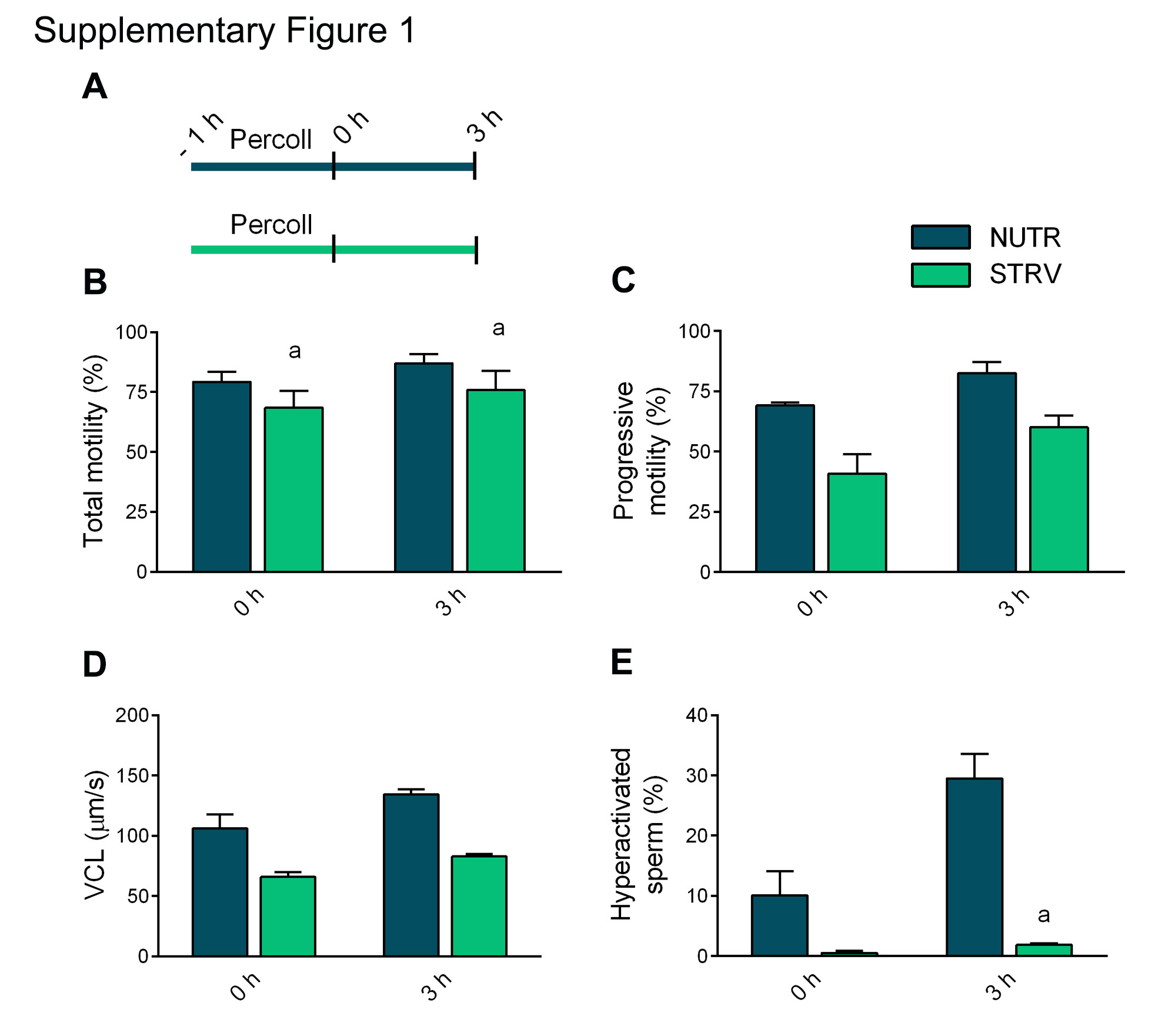

Supplement: Supplementary file 3 [file Image1.JPEG]

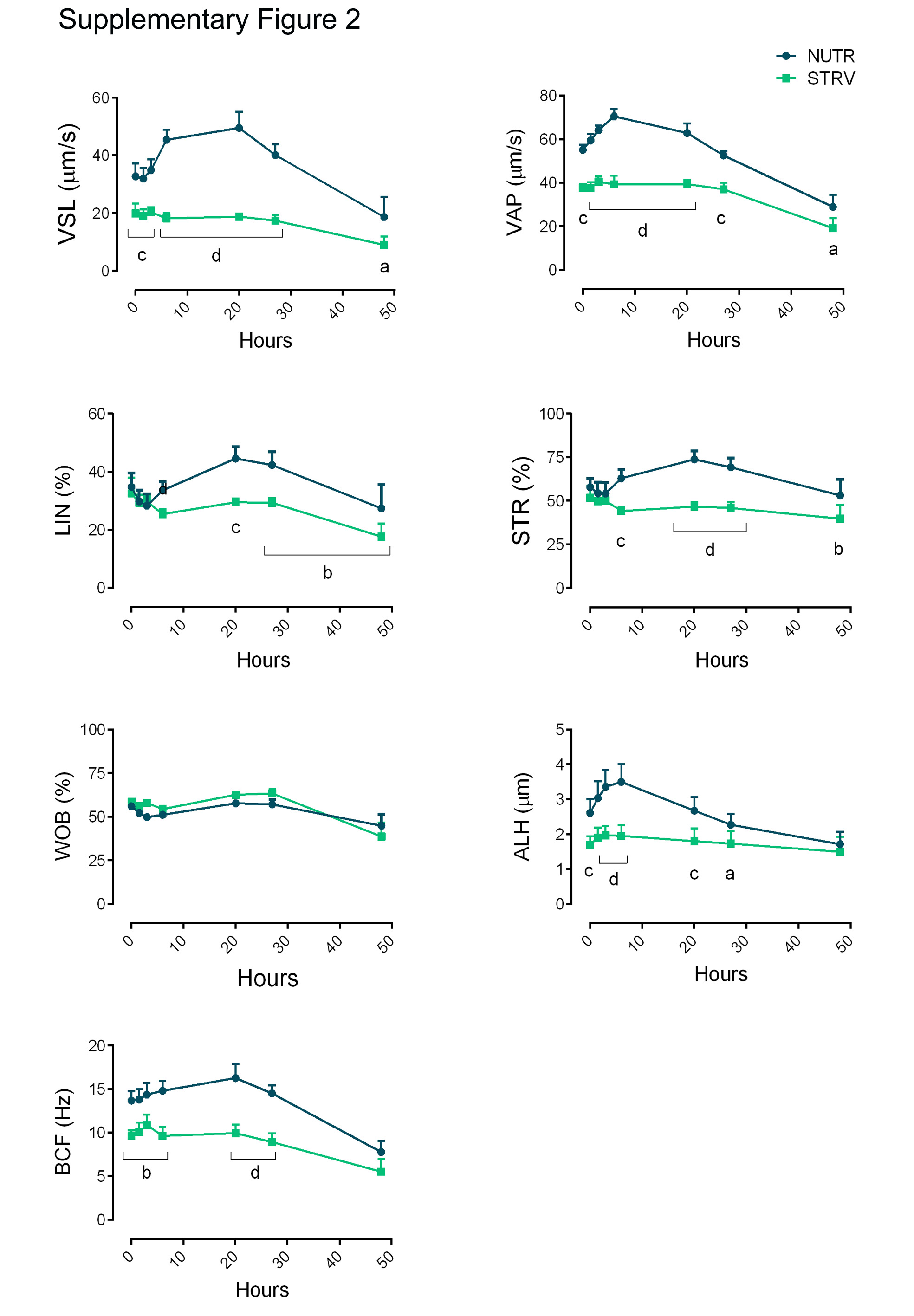

Supplement: Supplementary file 4 [file Image2.JPEG]
