# Supplementary material for: Human Sperm Remain Motile After a Temporary Energy Restriction but do Not Undergo Capacitation-Related Events
Source: Front Cell Dev Biol. 2021 Nov 12;9:777086. doi: 10.3389/fcell.2021.777086 (PMC8633110; doi:10.3389/fcell.2021.777086)
Supplement: Supplementary file 2 [file Table1.DOCX]

**Supplementary Table I:**

**Kinematic characteristics of human sperm evaluated after selection by density gradient centrifugation (0 h) or incubated for 3 h in medium with or without nutrients (NUTR and STRV, respectively).** Results are expressed as mean±SEM**,** n=3 experiments. ^a^ P<0.05, ^b^ P<0.01 vs. NUTR. Two-way ANOVA, and Bonferroni’s multiple comparison test.

|  | NUTR 0 h | STRV 0 h | NUTR 3 h | STRV 3 h |
| --- | --- | --- | --- | --- |
| VCL (μm/s) | 106±11 | 66±4 | 134±4 | 83±2 |
| VSL  (μm/s) | 40±3 | 23±4 ^b^ | 44±2 | 28±2 ^b^ |
| VAP (μm/s) | 59±3 | 37±3 | 74±2 | 48±1 ^a^ |
| LIN  (%) | 40±5 | 35±4 | 33±3 | 35±4 |
| STR  (%) | 66±3 | 59±5 | 61±2 | 58±4 |
| WOB  (%) | 57±4 | 57±2 | 53±3 | 58±2 |
| ALH  (μm) | 2.3±0.5 | 1.6±0.0 | 3.0±.0.1 | 1.9±0.1 |
| BCF  (Hz) | 17±1 | 12±1 | 17±1 | 14±1 |
